# Comparison of ceftriaxone versus ceftaroline in combination with ampicillin or penicillin against *Enterococcus faecalis*

**DOI:** 10.1128/spectrum.02718-24

**Published:** 2025-05-15

**Authors:** Olivia Gladys Funk, Ruhma Khan, Zeel Shah, Jaclyn A. Cusumano

**Affiliations:** 1 Long Island University 2045 , Brooklyn, New York, USA; University of Pittsburgh School of Medicine, Pittsburgh, Pennsylvania, USA

**Keywords:** *Enterococcus faecalis*, ceftaroline, penicillin, ceftriaxone, ampicillin

## Abstract

*Enterococcus faecalis* infective endocarditis treatment with ampicillin plus ceftriaxone has not changed the mortality rates by over 30%. We identified borderline-penicillin-resistant, ampicillin-susceptible *E. faecalis* (borderline-PRASEF; penicillin MIC 4–8 µg/mL, breakpoint ≤8 µg/mL) isolates that have decreased ampicillin-ceftriaxone activity, which is present in 25% of isolates in New York City. Alternatively, ceftaroline is more active against *E. faecalis* than ceftriaxone. We compared the activity of ampicillin or penicillin plus ceftriaxone or ceftaroline against nine borderline-PRASEF and seven penicillin-susceptible (penicillin MIC ≤2 µg/mL) *E. faecalis* isolates via 24 h time-kill assays. MICs were obtained via broth microdilution per CLSI. Ampicillin, penicillin, and ceftaroline were tested at subinhibitory concentrations (0.25 × MIC and 0.5 × MIC) and ceftriaxone at the free plasma steady-state concentration (17.2 µg/mL). All experiments were completed in duplicate with a starting inoculum of 10^6^ CFU/mL. After 24 h, antimicrobial activity was measured as ≥2-log_10_ CFU/mL decrease from the initial inoculum. Combinations were assessed for synergy (≥2-log_10_ CFU/mL decrease from the most active single agent). Ceftriaxone and ceftaroline MICs were more likely to be higher in borderline-PRASEF isolates compared to penicillin-susceptible isolates. Ceftaroline-based combinations more often demonstrated synergy and activity than ceftriaxone combinations. When assessed by penicillin MIC, ceftriaxone-based combinations less frequently demonstrated synergy and activity against borderline-PRASEF isolates compared to ceftaroline-based combinations. Ceftaroline-based combinations may be an effective alternative targeting *E. faecalis*, including borderline-PRASEF.

**IMPORTANCE:**

The preferred therapy for treatment of *Enterococcus faecalis* infective endocarditis is ampicillin plus ceftriaxone; however, there is a need for alternative treatments given the unchanged mortality rates exceeding 30%. Recent data show decreased ampicillin-ceftriaxone activity against borderline-penicillin-resistant, ampicillin-susceptible *E. faecalis* (borderline-PRASEF), which is present in 25% of isolates. Ceftaroline is an alternative cephalosporin that has been explored as it does not carry the risks ceftriaxone has to increase vancomycin resistance and *Clostridoides difficile* infection. Ceftaroline also provides saturation of both essential penicillin-binding protein (PBP) 4 and non-essential PBP2/3, whereas ceftriaxone only binds to PBP2/3. The activity of ceftaroline-based combinations against borderline-PRASEF is unknown. This study demonstrates the ability of ampicillin or penicillin plus ceftaroline to maintain activity against borderline-PRASEF where ampicillin or penicillin plus ceftriaxone combination activity is limited.

## INTRODUCTION

*Enterococcus faecalis* is the third most common infective endocarditis (IE) pathogen (1, 2); however, the introduction of transcatheter aortic valve implantation (TAVI) has increased the prevalence of *E. faecalis* IE. *E. faecalis* is the leading causative pathogen of TAVI-IE with an incidence of up to 40% (3, 4). Unfortunately, *E. faecalis* IE continues to have an unchanged mortality rate of over 30% (1, 5–7). Few advancements have been made in the current *E. faecalis* treatments as limited antimicrobials demonstrate activity due to intrinsic resistance (2).

The 2015 American Heart Association and Infectious Diseases Society of America IE guidelines recommend treating penicillin-susceptible *E. faecalis* IE with a combination therapy of penicillin or ampicillin plus gentamicin, or ampicillin plus ceftriaxone (2, 8, 9). Current practices have shifted toward adjunctive ceftriaxone over gentamicin due to increasing aminoglycoside resistance and associated gentamicin toxicities, but ampicillin plus ceftriaxone has not changed mortality or treatment failure rates (10–13). The 2023 European Society of Cardiology IE guidelines do not recommend penicillin-based treatments as ampicillin has MICs two to four times lower than penicillin (8). Penicillin is still considered to have a place in therapy due to the prolonged room temperature stability over ampicillin (14, 15). Penicillin plus ceftriaxone has also been assessed in small case series and cohort studies in which outcomes appear to be similar to ampicillin-ceftriaxone (14–18).

Although ampicillin plus ceftriaxone provides clinicians with an aminoglycoside-sparing regimen, ceftriaxone is not without limitations. Ceftriaxone can increase the risk of vancomycin-resistant enterococcus (VRE) colonization and *Clostridioides difficile* infections (7, 19). Additionally, *E. faecalis* is intrinsically resistant to ceftriaxone due to a lack of binding to essential penicillin-binding protein 4 (PBP4). However, ceftriaxone is theorized to provide a synergistic relationship with ampicillin, where ampicillin binds to essential PBP4 which prompts the upregulation of non-essential PBP2/3, the binding site for ceftriaxone (20).

Alternatively, ceftaroline binds to both essential PBP4 and non-essential PBP2/3 (21, 22). The additional PBP4 binding when combined with ampicillin or penicillin may provide improved bacterial killing compared to ampicillin plus ceftriaxone, which would be especially important against penicillin-resistant, ampicillin-susceptible *E. faecalis* (PRASEF) where PBP4 is upregulated (23, 24). Although PRASEF is uncommon in the United States (14), we identified borderline-PRASEF (penicillin MIC 4 to 8 µg/mL; CLSI breakpoint ≤8 µg/mL) isolates that have an incidence of 25% in New York City (25). This phenotype is less likely to demonstrate ampicillin-ceftriaxone *in vitro* synergy and bactericidal activity compared to penicillin-susceptible isolates (penicillin MIC ≤2 µg/mL) (26). The genetic implications of this effect have not been described, like in PRASEF isolates. Previous *in vitro* studies have demonstrated the synergistic effects of ampicillin plus ceftaroline (21, 27, 28), but only one study reports penicillin MICs and included five borderline-PRASEF, one penicillin-susceptible and one penicillin-resistant isolate, all of which ampicillin-ceftaroline maintained activity against (27). Furthermore, there are no studies that evaluate penicillin plus ceftaroline.

We hypothesize that ampicillin or penicillin plus ceftaroline will more frequently demonstrate *in vitro* synergy and antibacterial activity than ampicillin or penicillin plus ceftriaxone against *E. faecalis* isolates, including against borderline-PRASEF.

## RESULTS

### *Enterococcus faecalis* susceptibilities

Sixteen clinical *E. faecalis* blood isolates were tested against each antimicrobial combination. The ampicillin, penicillin, ceftriaxone, and ceftaroline MICs for each isolate are outlined in Table 1. All isolates were susceptible to ampicillin and penicillin per CLSI breakpoints (MIC ≤8 µg/mL). When separating isolates by penicillin MICs, nine were borderline-PRASEF and seven were penicillin-susceptible. No CLSI breakpoints are available for ceftriaxone and ceftaroline; however, borderline-PRASEF isolates were more likely to have ceftriaxone MICs ≥ 512 µg/mL and ceftaroline MICs ≥ 8 µg/mL compared to penicillin-susceptible isolates (penicillin MIC ≤2 µg/mL) (100% vs 28.6% [*P* = 0.005] and 100% vs 0% [*P* < 0.001], respectively).

**TABLE 1:** Minimum inhibitory concentrations (µg/mL) of *Enterococcus faecalis* clinical blood isolates by broth microdilution (*N* = 16)

Isolate ID	Ampicillin^*a*^	Penicillin^*b*^	Ceftriaxone^*c*^	Ceftaroline^*c*^
JH2-2	0.5	2	256	2
e2014	1	2	512	1
e2017	1	2	512	2
e2076	1	2	512	2
e2082	0.25	1	64	0.5
e2105	1	2	128	1
e2122	1	2	256	1
e2008	1	4	2,048	8
e2009	1	4	2,048	8
e2010	2	4	512	8
e2095	1	4	2,048	8
e2101	1	8	>2,048	64
e2110	1	4	2,048	8
e2121	1	4	>2,048	64
e2123	0.5	4	>2,048	64
e2143	2	8	>2,048	64

^*a*^ CLSI susceptibility breakpoint ≤8 µg/mL.

^*b*^ CLSI susceptibility breakpoint ≤8 µg/mL; borderline-PRASEF 4-8 µg/mL.

^*c*^ No CLSI MIC breakpoints listed for ceftriaxone or ceftaroline against *E. faecalis*.

### Time-kill analyses

Each antimicrobial alone did not achieve ≥2-log_10_CFU/mL kill, except three borderline-PRASEF isolates (18.8%) against ampicillin 0.5 × MIC. Thus, the median log_10_CFU/mL change for all borderline-PRASEF isolates against ampicillin 0.5 × MIC was −1.67 (interquartile range [IQR], −2.10 to 0.99) compared to penicillin-susceptible isolates which was 0.74 (IQR, 0.22–1.20; *P* = 0.223). Figures S1 and S2 demonstrate the time-kill curves of each individual isolate.

**Fig 1:**
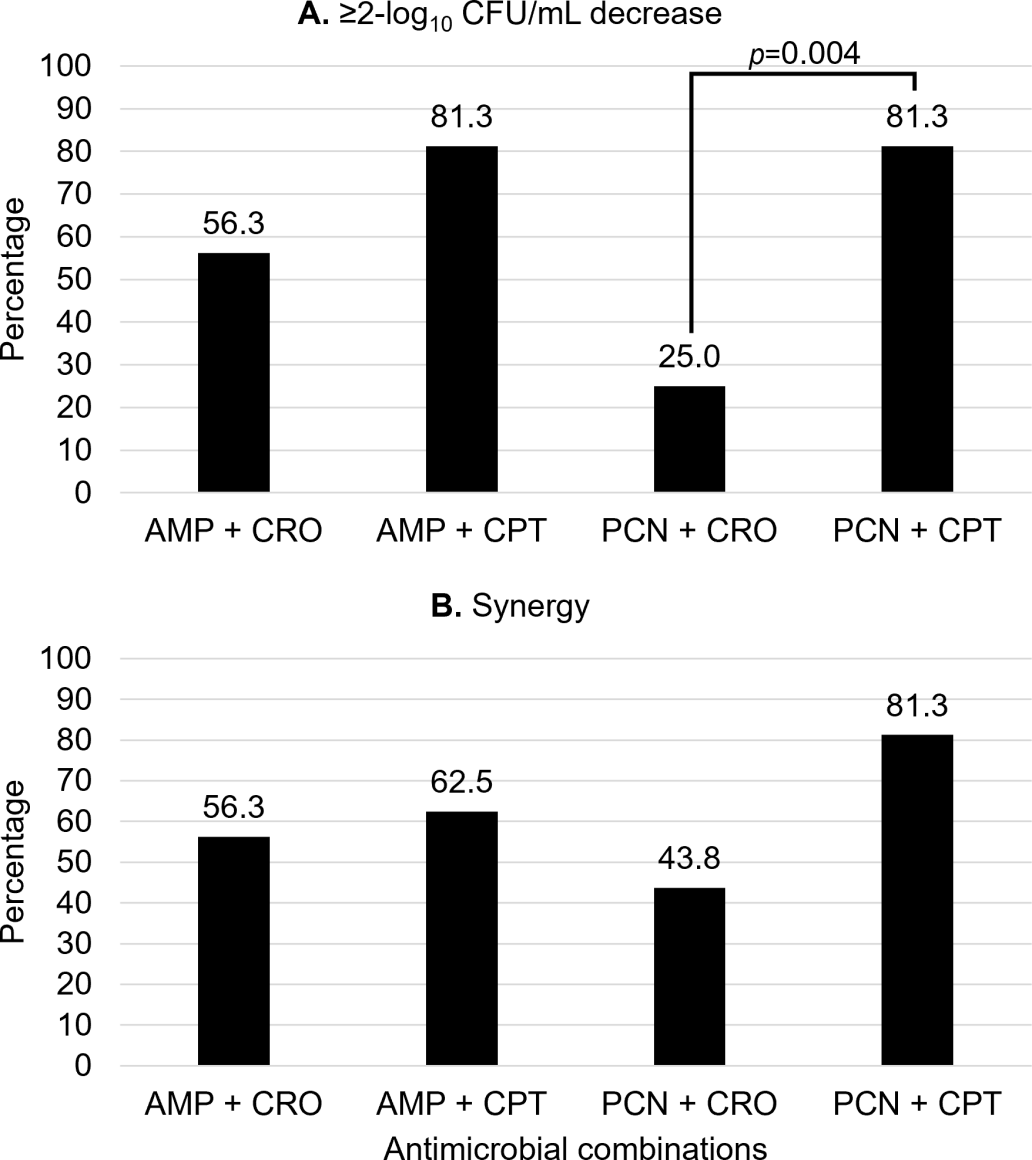
Overall activity of ceftriaxone versus ceftaroline combinations against clinical *Enterococcus faecalis* blood isolates via 24 h time-kill assays (*N* = 16). (**A**) Percentage of isolates that the antimicrobial combinations achieved ≥2-log_10_CFU/mL decrease from the initial inoculum. (**B**) The percentage of isolates that the antimicrobial combinations achieved synergy. Data reported are if at least one combination tested (i.e., ampicillin or penicillin 0.25 × MIC and 0.5 × MIC, plus ceftriaxone 17.2 µg/mL or ceftaroline 0.25 × MIC and 0.5 × MIC) achieved the desired endpoint. Abbreviations: AMP, ampicillin; CPT, ceftaroline; CRO, ceftriaxone; and PCN, penicillin. Ceftriaxone was tested at the free plasma steady-state concentration (*f*Cp_ss_=17.2 µg/mL) based on population pharmacokinetic data for a 2 g IV q12h regimen.

Overall, ceftaroline combinations more often achieved ≥2-log_10_CFU/mL kill compared to ceftriaxone combinations (Fig. 1A). Penicillin-ceftaroline combinations were significantly more likely to achieve ≥2-log_10_CFU/mL kill compared to penicillin-ceftriaxone combinations (81.3% vs 25.0%, respectively; *P* = 0.004). Ampicillin-ceftaroline combinations were numerically more likely to achieve ≥2-log_10_CFU/mL kill compared to penicillin-ceftriaxone combinations (81.3% vs 56.3%; *P* = 0.252). Differences in synergy between ceftriaxone and ceftaroline were statistically similar but were higher among ceftaroline combinations (Fig. 1B).

**Fig 2:**
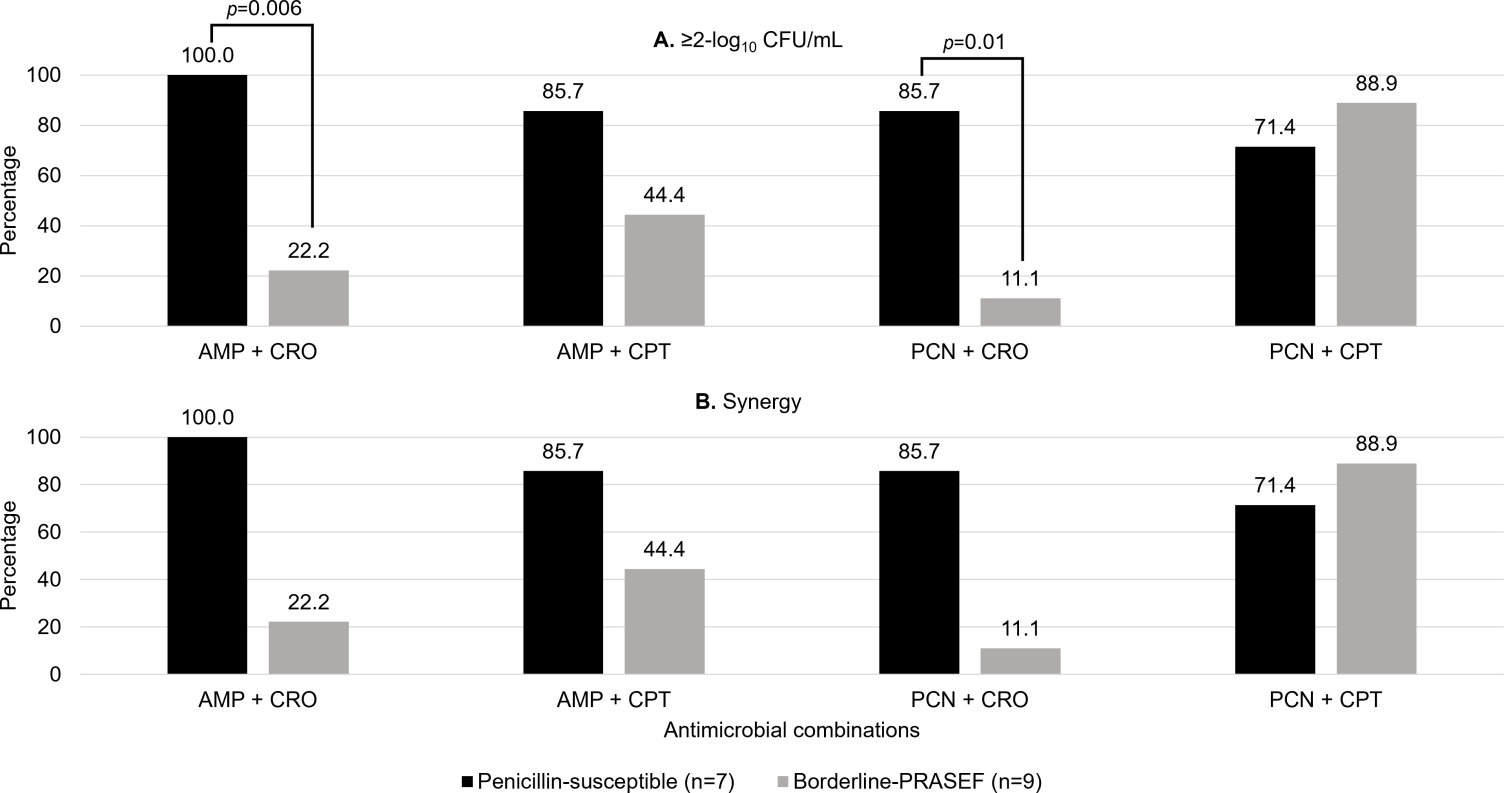
Activity of ceftriaxone and ceftaroline combinations against penicillin-susceptible (*n* = 7) versus borderline-PRASEF (*n* = 9) clinical blood isolates via 24 h time-kill assays. (**A**) Percentage of isolates that the antimicrobial combinations achieved ≥2-log_10_CFU/mL decrease from the initial inoculum. (**B**) The percentage of isolates that the antimicrobial combinations achieved synergy. Data reported are if at least one combination tested (i.e., ampicillin or penicillin 0.25 × MIC and 0.5 × MIC, plus ceftriaxone 17.2 µg/mL or ceftaroline 0.25 × MIC and 0.5 × MIC) achieved the desired endpoint. Abbreviations: AMP, ampicillin; CPT, ceftaroline; CRO, ceftriaxone; PCN, penicillin; and PRASEF, penicillin-resistant, ampicillin-susceptible. Penicillin-susceptible: MIC ≤2 µg/mL. Borderline-PRASEF: MIC 4-8 µg/mL. ^a^Ceftriaxone was tested at the free plasma steady-state concentration (*f*Cp_ss_=17.2 µg/mL) based on population pharmacokinetic data for a 2 g IV q12h regimen.

When stratified by the penicillin MIC, ampicillin-ceftriaxone and penicillin-ceftriaxone combinations were statistically significantly less likely to achieve ≥2-log_10_CFU/mL kill and synergy against borderline-PRASEF compared to penicillin-susceptible isolates (Fig. 2A and B). Ampicillin-ceftaroline and penicillin-ceftaroline combinations demonstrated statistically similar ≥2-log_10_CFU/mL kill and synergy; however, penicillin-ceftaroline combinations were the most active against borderline-PRASEF.

As four isolates had much higher ceftaroline MICs of 64 µg/mL (e2101, e2121, e2123, and e2143), we did a post-hoc analysis comparing isolates with a ceftaroline MIC of 64 µg/mL to isolates with ceftaroline MICs ≤ 8 µg/mL against ceftaroline combinations (Table 2). While differences between the two groups were not statistically significant, there was a general trend of decreased ≥2-log_10_CFU/mL kill and synergy of combinations against isolates with a higher ceftaroline MIC of 64 µg/mL.

**TABLE 2:** Activity of antimicrobial combinations stratified by ceftaroline minimum inhibitory concentration against *Enterococcus faecalis* isolates via 24 h time-kill assays^*a*^

Ampicillin or penicillin	Ceftaroline	Ceftaroline MIC ≤8 µg/mL *n* = 12	Ceftaroline MIC 64 µg/mL *n* = 4	*P*-value
Synergy, no. (%)				
Ampicillin 0.25× MIC +	Ceftaroline 0.25× MIC	2 (16.7)	0	1
	Ceftaroline 0.5× MIC	7 (58.3)	1 (25.0)	0.569
Ampicillin 0.5× MIC +	Ceftaroline 0.25× MIC	4 (33.3)	0	0.516
	Ceftaroline 0.5× MIC	7 (58.3)	1 (25.0)	0.569
Penicillin 0.25× MIC +	Ceftaroline 0.25× MIC	4 (33.3)	0	0.516
	Ceftaroline 0.5× MIC	6 (50.0)	1 (25.0)	0.585
Penicillin 0.5× MIC +	Ceftaroline 0.25× MIC	8 (66.7)	2 (50.0)	0.604
	Ceftaroline 0.5× MIC	9 (75.0)	3 (75.0)	1
≥2-log_10_CFU/mL decrease, no. (%)				
Ampicillin 0.25× MIC +	Ceftaroline 0.25× MIC	1 (8.3)	0	1
	Ceftaroline 0.5× MIC	8 (66.7)	1 (25.0)	0.262
Ampicillin 0.5× MIC +	Ceftaroline 0.25× MIC	6 (50.0)	0	0.234
	Ceftaroline 0.5× MIC	10 (83.3)	2 (50.0)	0.245
Penicillin 0.25× MIC +	Ceftaroline 0.25× MIC	3 (25.0)	0	0.529
	Ceftaroline 0.5× MIC	4 (33.3)	0	0.516
Penicillin 0.5× MIC +	Ceftaroline 0.25× MIC	7 (58.3)	1 (25.0)	0.569
	Ceftaroline 0.5× MIC	10 (83.3)	2 (50.0)	0.245

^*a*^ IQR, interquartile range.

## DISCUSSION

Among 16 clinical *E. faecalis* blood isolates tested, ampicillin or penicillin plus ceftaroline was more active than when combined with ceftriaxone. Ceftaroline-based combinations maintained synergy and activity against borderline-PRASEF isolates, whereas ceftriaxone-based combinations had decreased synergy and activity.

Ampicillin plus ceftriaxone is currently the first-line therapy for *E. faecalis* IE (2, 8); however, the clinical impact of penicillin susceptibility has not been clearly elucidated. The *in vitro* and *in vivo* studies that introduced ampicillin-ceftriaxone as a potential treatment option did not report penicillin MICs (20, 29, 30). Later clinical studies that enabled the inclusion of ampicillin plus ceftriaxone in IE guidelines and recent retrospective studies also did not include penicillin susceptibilities (10, 31–34).

We recently compared ampicillin plus ceftriaxone to penicillin plus ceftriaxone, given the favorable stability of penicillin over ampicillin (26). Subsequently, we identified that both ampicillin and penicillin plus ceftriaxone were less commonly synergistic and bactericidal against borderline-PRASEF isolates. The prevalence of borderline-PRASEF is not well described due to limited reporting of penicillin susceptibility; however, we identified that 25% of New York City isolates were borderline-PRASEF. We acknowledge that the borderline-PRASEF strains included in this study from New York City may be clonal, so we ensured the inclusion of isolates from Detroit, MI, which demonstrated similar results.

Ampicillin plus ceftriaxone is theorized to be synergistic against *E. faecalis* due to the total saturation of PBPs, which are the backbone of peptidoglycan synthesis to build a stable cell wall. Ampicillin and penicillin target PBP4, which is essential to *E. faecalis* survival, whereas ceftriaxone targets PBP2/3, which is non-essential for survival. PRASEF has been shown to increase PBP4 expression, contributing to decreased β-lactam susceptibilities (23, 24). Borderline-PRASEF similarly has been shown to increase PBP4 expression but to a lesser extent than PRASEF (35). Isolates overexpressing PBP4 have also been associated with high ceftriaxone MICs and decreased ampicillin-ceftriaxone activity (36), which is similar to our observations in our borderline-PRASEF isolates (26). Therefore, due to ceftaroline binding to both PBP4 and PBP2/3 (20, 22), we hypothesized that ampicillin or penicillin plus ceftaroline will maintain activity against borderline-PRASEF isolates. Further studies are warranted to determine the PBP genetic mechanisms underlying the improved activity of ceftaroline over ceftriaxone.

Ampicillin plus ceftaroline has been previously compared to ampicillin-ceftriaxone *in vitro* only; however, only one study reported penicillin MICs (27). Based on the reported MICs, one penicillin-susceptible, five borderline-PRASEF, and one PRASEF isolate were tested using 24 h time-kill assays, which showed that ampicillin-ceftaroline combinations demonstrated greater activity, synergy, and bactericidal activity (≥3-log_10_CFU/mL reduction from baseline) against borderline-PRASEF isolates than ampicillin-ceftriaxone (27). Other studies not including penicillin MICs may be able to be assumed based on ceftriaxone and ceftaroline MICs. While MIC breakpoints have not been established for either ceftriaxone or ceftaroline against *E. faecalis*, higher MICs may help indicate the presence of a borderline-PRASEF isolate. Our included borderline-PRASEF isolates had higher MICs (ceftaroline MIC range, 8–64 µg/mL; ceftriaxone MIC range 512 to >2048 µg/mL) compared to penicillin-susceptible isolates. An *in vitro* checkerboard analysis of 21 *E. faecalis* isolates observed synergy in 95% of isolates against ampicillin-ceftaroline versus 81% against ampicillin-ceftriaxone (21). The isolates included had lower ceftaroline and ceftriaxone MICs (range: 0.125–8 µg/mL and 2 to >512 µg/mL, respectively), likely implying few included borderline-PRASEF, which may explain the observed greater synergy rate than in our study. Differences in results may also be due to the use of fractional inhibitory concentration index (FICI), which is not an accurate predictor of synergy due to varying methods of calculation, making it difficult to interpret findings (37).

In addition, two other studies assessing dosing strategies *in vitro* did not report penicillin MICs (28, 38). These studies were both pharmacodynamic models and reported greater activity with ampicillin-ceftaroline than ampicillin-ceftriaxone against *E. faecalis* isolates at standard dosing. Between these two studies, it can be assumed that two of the five isolates were borderline-PRASEF isolates based on the ceftriaxone and ceftaroline MIC trends. In both of these isolates, ampicillin-ceftaroline demonstrated the most activity, whereas ampicillin-ceftriaxone demonstrated slightly less activity, further supporting ceftaroline usage against borderline-PRASEF isolates.

While ampicillin-ceftriaxone is the mainstay of therapy against *E. faecalis*, ampicillin stability is variable, emphasizing the importance of penicillin to be further studied. To our knowledge, this is the first study to evaluate the activity and synergy of penicillin-ceftaroline against *E. faecalis*. There are few studies that evaluated the *in vitro* activity and clinical efficacy of penicillin-ceftriaxone as an alternative to ampicillin-ceftriaxone against *E. faecalis* IE (14–18). Reported mortality rates were up to 20%; however, the population sizes were small. But despite this, these mortality rates are similar to the 30% mortality seen with ampicillin-ceftriaxone treatment. One of the studies tested the synergy of benzylpenicillin plus ceftriaxone in an *in vitro* checkerboard assay against six isolates, in which synergy was attained in three isolates (17). All of the isolates in the study were penicillin-susceptible.

An inherent limitation of time-kill assays is the static nature, as only a time- or concentration-dependent effect can be observed. Time-kill assays are unable to mimic physiologic antimicrobial pharmacokinetics (PK). In the present study, we aimed to assess each antimicrobial at physiologically achievable concentrations in the human body. Ampicillin and penicillin were tested at concentrations subinhibitory to the MIC as free plasma steady-state levels (*f*Cp_SS_) are much higher than the MICs which would lead to complete isolate eradication *in vitro*. Ceftriaxone, however, was tested at the free plasma steady-state concentration as MICs are much higher than physiologic concentrations due to intrinsic resistance. Finally, ceftaroline was tested at subinhibitory MICs, after comparing ceftaroline activity at subinhibitory MICs to *f*Cp_SS_ (11.1 µg/mL) against two isolates (e2122: penicillin MIC, 2 µg/mL, ceftaroline MIC, 1 µg/mL; e2095: penicillin MIC, 4 µg/mL, ceftaroline MIC, 8 µg/mL). We found that *f*Cp_SS_ resulted in complete eradication of e2122 and nearly 2-log_10_CFU/mL kill against e2095 (−1.98 ± 0.09 log _10_ CFU/mL), which would make it hard to determine activity in combination with ampicillin or penicillin. Conversely, subinhibitory concentrations resulted in growth. However, after the experiment design, four isolates had a ceftaroline MIC of 64 µg/mL, which would result in subinhibitory concentrations that are greater than the *f*Cp_SS_ (11.1 µg/mL vs 0.25 × MIC, 16 µg/mL or 0.5 × MIC, 32 µg/mL). As a result, we did a post-hoc analysis comparing ceftaroline combination activity against isolates with a MIC of 64 µg/mL versus MICs ≤8 µg/mL. We found a trend toward decreased activity and synergy in isolates with an MIC of 64 µg/mL. The results were not statistically significant likely due to a small sample size of isolates with an MIC of 64 µg/mL. Furthermore, our study is limited by the lack of genetic testing which leaves the question of isolate clonality and the PBP mechanism driving improved ceftaroline activity unknown.

While we observed decreased activity of ceftaroline combinations against isolates with ceftaroline MICs of 64 µg/mL, it is important to consider PKPD dose optimization, especially as all these isolates are borderline-PRASEF and standard-of-care ampicillin-ceftriaxone also has decreased activity. In general, the PKPD target for β-lactams is time-dependent (*f*T >MIC); however, in the setting of *E. faecalis* treatment, ceftriaxone is considered a synergistic agent achieving 0% *f*T >MIC due to MICs much higher than physiologic concentrations (*f*Cp_SS_, 17.2 mg/L). Thus, suggesting that both ceftriaxone and ceftaroline as synergistic agents may exhibit a different PKPD parameter than *f*T >MIC. More studies are needed to evaluate the true PKPD target and ceftaroline dosing to overcome isolates with higher ceftaroline MICs of 64 µg/mL.

Ampicillin or penicillin plus ceftaroline against *E. faecalis* could be a promising alternative to ampicillin-ceftriaxone, especially against borderline-PRASEF. Our study stresses the importance of reporting penicillin MICs, as we demonstrated decreased activity and synergy of ampicillin- and penicillin-ceftriaxone combinations against borderline-PRASEF isolates. While ceftaroline and ceftriaxone combinations are comparable against penicillin-susceptible isolates, when treating a borderline-PRASEF isolate, alternative therapy with ampicillin-ceftaroline may be warranted over ampicillin-ceftriaxone. Additionally, penicillin-based combinations may be a promising alternative to ampicillin-based combinations against *E. faecalis*, given improved stability at room temperature; however, ceftaroline’s room temperature stability is similar to ampicillin. The clinical applicability and implications of these *in vitro* findings are still warranted.

## MATERIALS AND METHODS

### Bacterial isolates

A total of 16 *E. faecalis* isolates were randomly selected for inclusion, 15 of which were unique clinical blood isolates from two different health systems (New York, NY and Detroit, MI) and one well-characterized, penicillin-susceptible isolate, JH2-2 (23). Five of the clinical isolates were from Detroit, MI, and were previously described against ampicillin-ceftriaxone and penicillin-ceftriaxone (26). Per broth microdilution (BMD), all isolates were susceptible to ampicillin and penicillin per CLSI breakpoints (penicillin MIC ≤8 µg/mL) (39); however, nine isolates were borderline-PRASEF (penicillin MIC 4–8 µg/mL) and were selected for comparison. All isolates were stored at −80°C in tryptic soy broth plus glycerol (CryoCare, Stamford, TX) and were sub-cultured once on brain heart infusion agar (BHIA) for 18–24  h at 35°C prior to each experiment.

### Antimicrobials and media

Antimicrobial active pharmaceutical ingredients used included ampicillin sodium (Sigma-Aldrich Inc., Saint Louis, MO, product number: A0166), penicillin G potassium (Sigma-Aldrich Inc., product number: 46609), and ceftriaxone sodium (Sigma-Aldrich Inc., product number: PHR1382) which were stored at 4°C and ceftaroline dihydrochloride (AbbVie Inc., Chicago, IL) which was stored at −20°C. Antimicrobial solutions were made fresh for each experiment. BMD and time-kill assays were performed using cation-adjusted (calcium, 25  µg/mL; magnesium, 12.5  µg/mL) Mueller-Hinton broth (MHB; BD Difco, Sparks, MD). Isolate sub-cultures and viable cell counts to verify inoculums were plated to BHIA (BD Difco, Sparks, MD).

### Antimicrobial susceptibility testing

MICs were determined in duplicate for each isolate against penicillin, ampicillin, ceftriaxone, and ceftaroline via BMD per CLSI standards (39). Quality control strains were utilized to confirm antimicrobial activity, ATCC 29213 *Staphylococcus aureus* for ceftriaxone and ceftaroline, and ATCC 29212 *E. faecalis* for penicillin and ampicillin. Plates were incubated at 35°C and read at 20 h. Isolates were categorized as susceptible or resistant to ampicillin or penicillin based on CLSI breakpoints (susceptible, ≤8 µg/mL; resistant, ≥16 µg/mL). Susceptible penicillin isolates were further categorized as borderline-PRASEF if there was an elevated penicillin MIC of 4–8 µg/mL or penicillin-susceptible with an MIC ≤2 µg/mL. CLSI currently does not publish MIC breakpoints for ceftriaxone and ceftaroline against *E. faecalis*. MICs were repeated if the result was a range or there were skipped wells.

### Time-kill assays

Each isolate was tested against ampicillin, penicillin, ceftriaxone, and ceftaroline alone as well as ampicillin or penicillin plus ceftriaxone or ceftaroline combinations via 24 h static time-kill assays, in duplicate. Twelve-well plates were utilized with a final volume of 2 mL in each well. Each *E. faecalis* isolate was prepared as a 0.5 McFarland for a target starting inoculum of 10^6^ CFU/mL. Plates were incubated at 35°C and placed on an orbital shaker at 50 rotations per minute.

Ampicillin, penicillin, and ceftaroline were tested at subinhibitory concentrations (0.25 × MIC and 0.5 × MIC), whereas ceftriaxone was tested at the *f*Cp_SS_ of 17.2 µg/mL based on population PK for a 2 g intravenous injection every 12 h regimen (t_1/2_=7.2 h, *f*C_max_ = 28.9  µg/mL), as subinhibitory concentrations are not physiologically achievable due to the enterococcus intrinsic resistance, as previously described (40, 41). Samples were taken at 0, 4, and 24 h and subsequently diluted in normal saline and plated to BHIA. Samples were incubated for 18–24 h at 35°C to obtain viable cell counts. The lower limit of detection was 2-log_10_CFU/mL. Median log_10_CFU/mL change and ≥2-log_10_CFU/mL kill from initial inoculum was measured at 24 h. A ≥2-log_10_ decrease in CFU/mL was chosen as ≥1-log_10_ kill and stasis would not be sufficient to reduce bacterial burden in endocarditis. In animal models, ≥2-log_10_ kill has been shown to improve outcomes and, therefore, was selected over traditional bactericidal ≥3-log_10_ kill (42–45). Synergy was measured as a ≥2-log_10_CFU/mL decrease from the most active single agent at 24 h.

### Statistical analysis

All statistical analyses were completed using R Studio (R version 4.1.2 [http://www.R-project.org/]). Differences in categorical variables were assessed by Fisher’s exact test, and continuous variables were assessed with Wilcoxon Rank Sum. In both cases, statistical significance was determined by a two-sided *P*-value of <0.05.

## SUPPLEMENTAL MATERIAL

Fig. S1 spectrum.02718-24-s0001.tiff Supplemental figure 1.

Fig. S2 spectrum.02718-24-s0002.tiff Supplemental figure 2.

ASM does not own the copyrights to Supplemental Material that may be linked to, or accessed through, an article. The authors have granted ASM a non-exclusive, world-wide license to publish the Supplemental Material files. Please contact the corresponding author directly for reuse.
