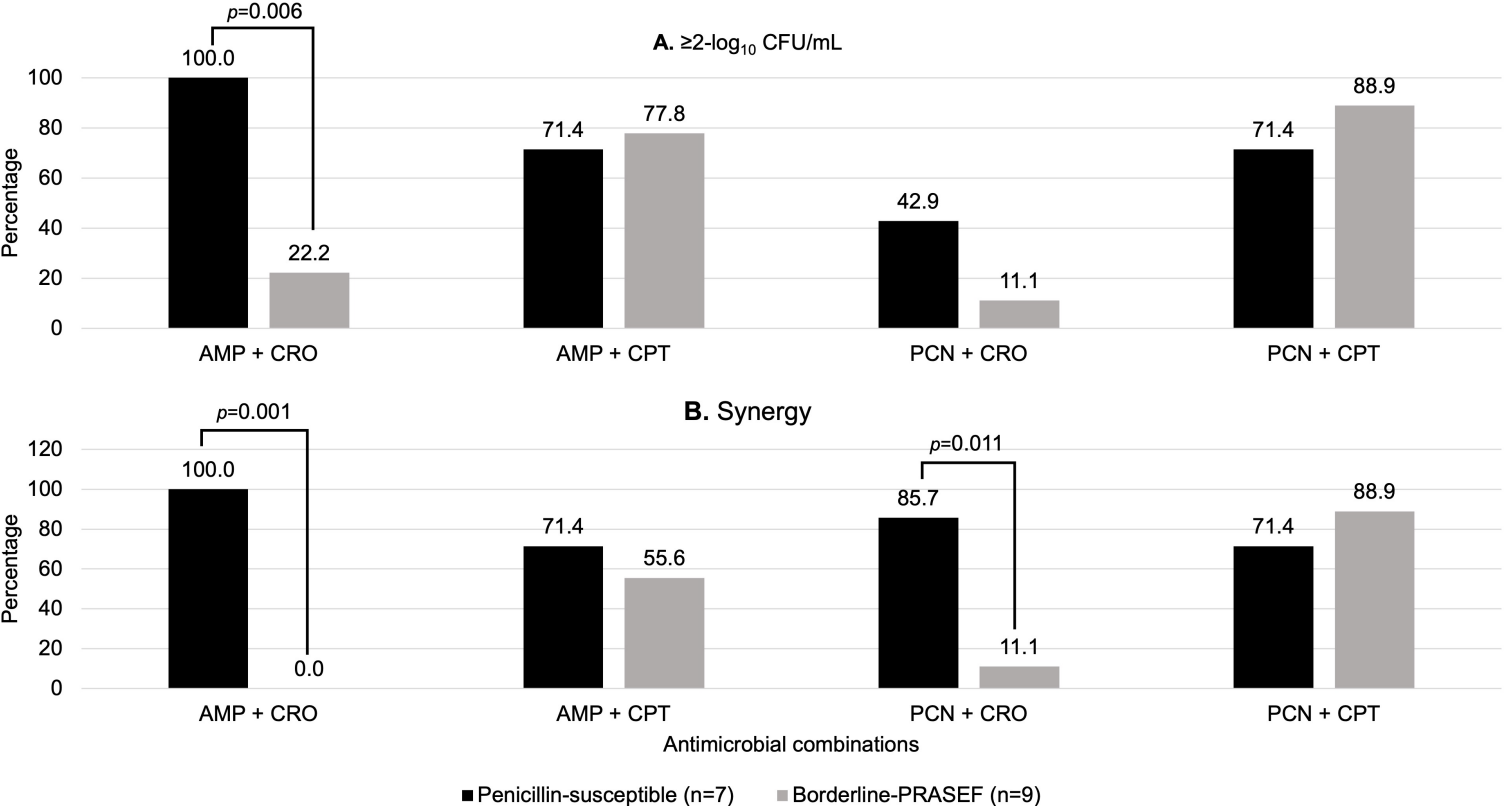# Correction for Funk et al., “Comparison of ceftriaxone versus ceftaroline in combination with ampicillin or penicillin against *Enterococcus faecalis*”

**DOI:** 10.1128/spectrum.02176-25

**Published:** 2025-12-03

**Authors:** Olivia Gladys Funk, Ruhma Khan, Zeel Shah, Jaclyn A. Cusumano

## AUTHOR CORRECTION

Volume 13, no. 6, e0271824, 2025, https://doi.org/10.1128/spectrum.02718-24. Page 3, Paragraph 3: “…were statistically significantly less likely…” should read “…were less likely…”

Page 5: Fig. 2 should appear as shown in this correction. The percentages displayed in Fig. 2A were adjusted for ampicillin-ceftaroline and penicillin-ceftriaxone and in Fig. 2B were adjusted for ampicillin-ceftriaxone and ampicillin-ceftaroline. These errors occurred due to an incorrect data entry in the Excel file with the corresponding figure. However, they do not affect the conclusions of the manuscript.

**Fig 2:**