# Supplementary figures and images for: Comparison of ceftriaxone versus ceftaroline in combination with ampicillin or penicillin against Enterococcus faecalis
Source: Microbiol Spectr. 2025 May 15;13(6):e02718-24. doi: 10.1128/spectrum.02718-24 (PMC12131770; doi:10.1128/spectrum.02718-24)

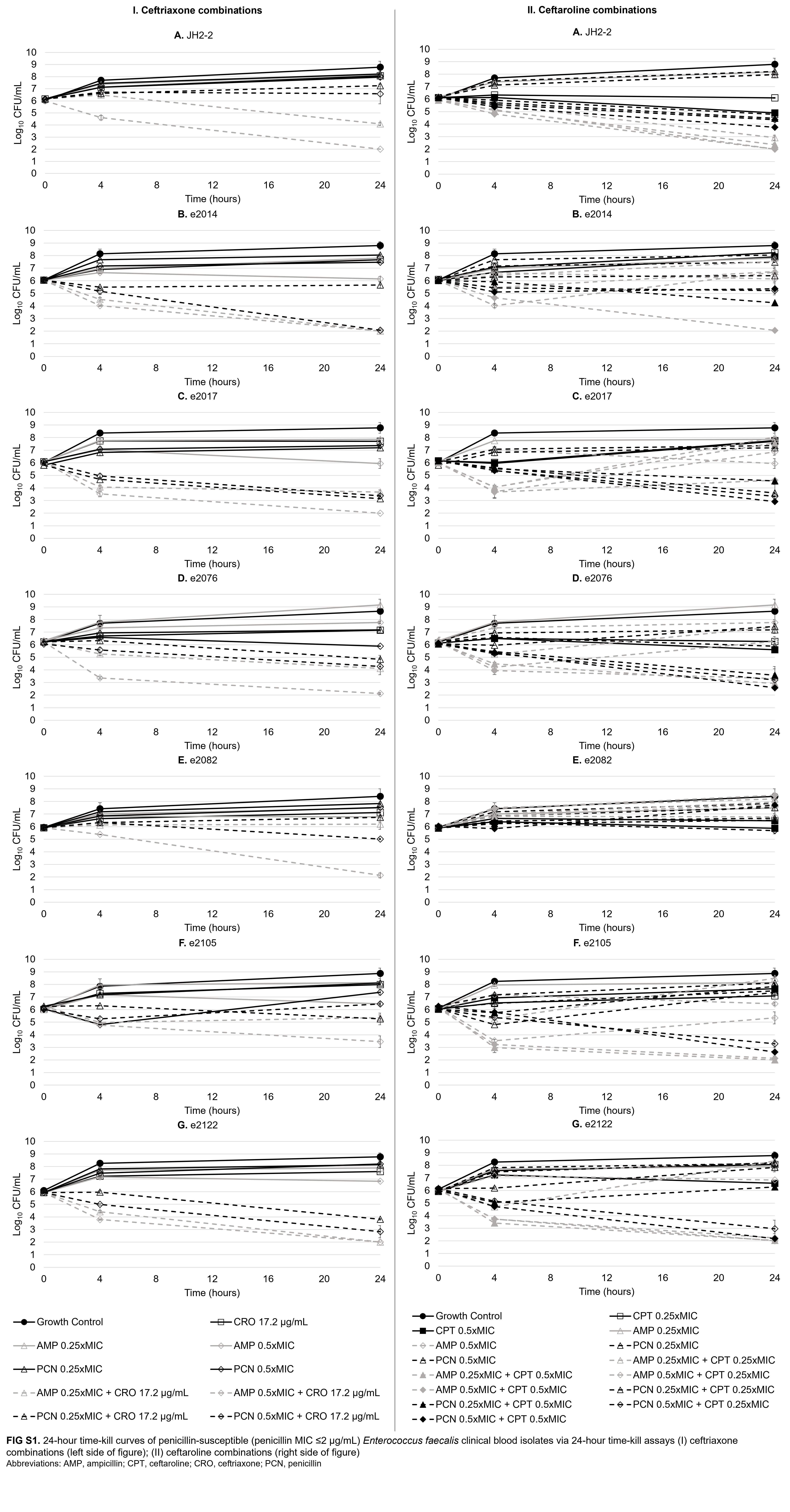

Supplement: Fig. S1 — Supplemental figure 1. [file spectrum.02718-24-s0001.tiff]

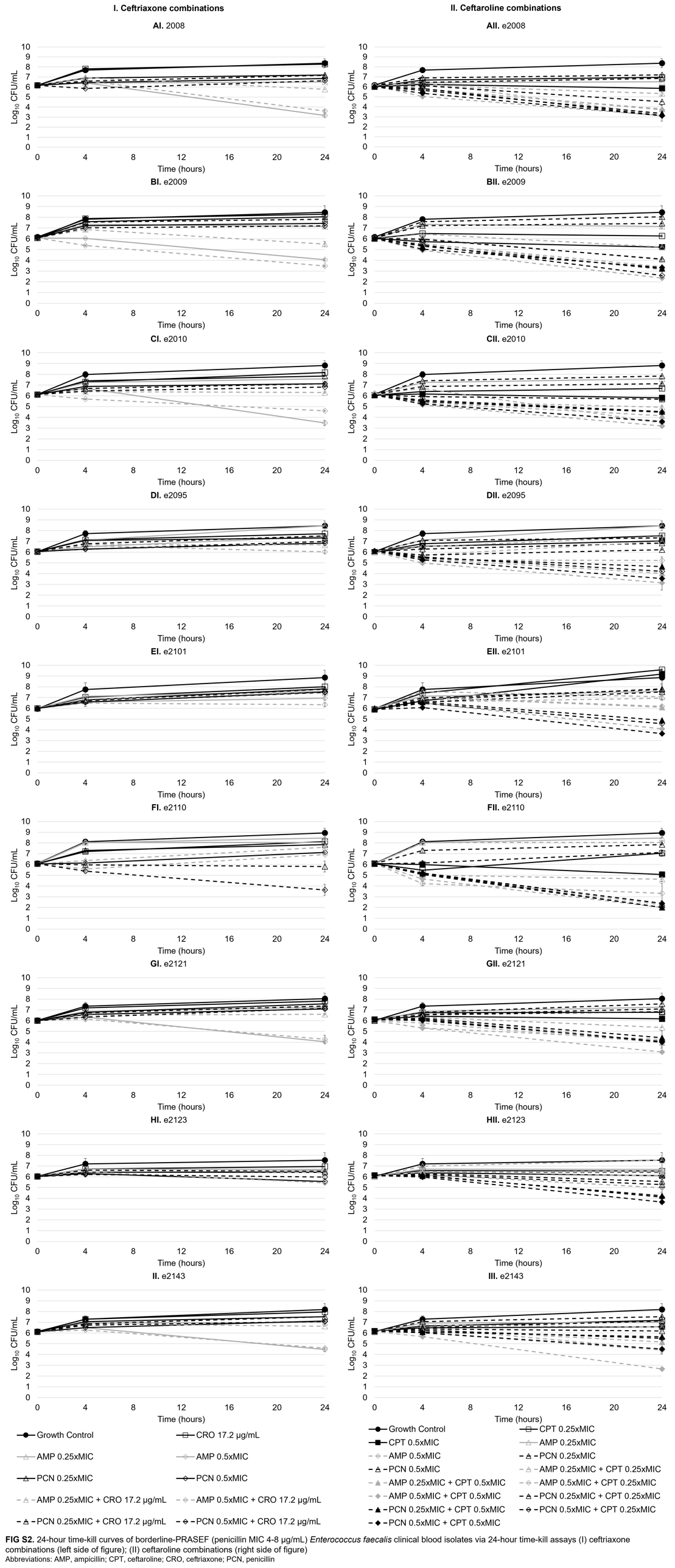

Supplement: Fig. S2 — Supplemental figure 2. [file spectrum.02718-24-s0002.tiff]
